# Restoring visual function to the blind retina with a potent, safe and long-lasting photoswitch

**DOI:** 10.1038/srep45487

**Published:** 2017-04-13

**Authors:** Ivan Tochitsky, Jay Trautman, Nicholas Gallerani, Jonatan G. Malis, Richard H. Kramer

**Affiliations:** 1 Department of Molecular and Cell Biology, University of California, Berkeley, CA 94720, USA; 2 Photoswitch Biosciences, Inc., Menlo Park, CA, USA

## Abstract

Photoswitch compounds such as DENAQ confer light-sensitivity on endogenous neuronal ion channels, enabling photocontrol of neuronal activity without genetic manipulation. DENAQ treatment restores both retinal light responses and visual behaviors in rodent models of Retinitis pigmentosa. However, retinal photosensitization requires a high dose of DENAQ and disappears within several days after treatment. Here we report that BENAQ, an improved photoswitch, is 20-fold more potent than DENAQ and persists in restoring visual responses to the retina for almost 1 month after a single intraocular injection. Studies on mice and rabbits show that BENAQ is non-toxic at concentrations 10-fold higher than required to impart light-sensitivity. These favorable properties make BENAQ a potential drug candidate for vision restoration in patients with degenerative blinding diseases.

Retinitis pigmentosa (RP) and age-related macular degeneration (AMD) are degenerative diseases involving the loss of rod and cone photoreceptors, leading to visual impairment and in some cases complete blindness. Several technologies are being pursued as potential vision restoring treatments for advanced RP or AMD. Optoelectronic retinal prosthetics can be surgically implanted in the eye where they electrically stimulate the retinal neurons that survive after photoreceptor death, restoring some visual perception to blind patients^1^. Stem cell-derived photoreceptors can be transplanted into the retina where they can restore some visual function in animal models of RP^2^. Viral expression of optogenetic tools, including light sensitive microbial opsins, can restore electrophysiological and behavioral responses to light in mouse models of RP^3^,^4^. These technologies have produced promising results, but each involves invasive and/or irreversible manipulations. While the permanence of optoelectronic, stem cell, or optogenetic interventions could be favorable in the absence of complications, any deleterious effects of these treatments might be difficult or impossible to reverse.

To avoid these potential issues, we have developed an alternative strategy for vision restoration using drug-like small molecules. We have created a series of synthetic azobenzene photoswitch compounds that confer light sensitivity onto endogenous voltage-gated cation channels without requiring genetic manipulation, enabling photocontrol of electrical excitability in retinal neurons^5^,^6^,^7^. Retinal light responses mediated by a photoswitch named DENAQ are elicited by stimuli similar in intensity and spectrum to ordinary daylight and the photosensitization is selective for blind retinas with degenerated photoreceptors^6^,^7^,^8^. Photosensitizing doses of DENAQ also have no apparent toxic effect on the mouse retina^6^,^8^, although the compound’s safety in large animal eyes is unknown.

Taken together, these features make DENAQ an intriguing candidate as a vision-restoring drug for advanced RP and AMD. However, there are some important limitations: a high concentration of DENAQ (300 μM–1 mM) is required for retinal photosensitization, raising the risk of off-target effects. Furthermore, photosensitization declines with a half-life of ~2 days, requiring an unacceptably frequent treatment schedule. Here, we present BENAQ, an improved photoswitch that is potent, long-lasting, and safe in large animal eyes. The action of BENAQ is also selective for blind retinas in animals suffering from retinal degeneration while having no apparent effect on retinas from healthy sighted animals. Together, these properties make BENAQ potentially more suitable for future clinical use.

## Results

### BENAQ restores fast, spatially precise light responses to the blind mouse retina

**Figure 1:**
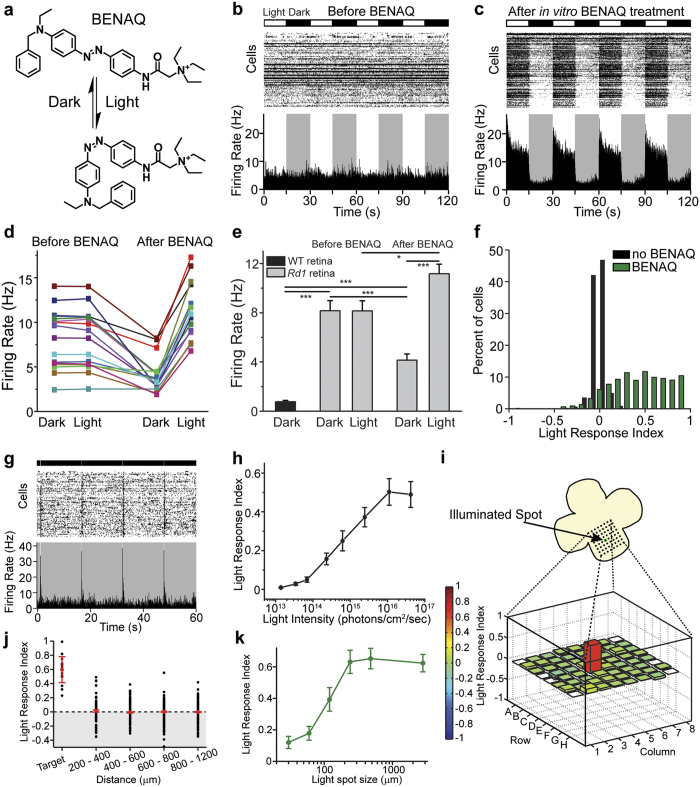
BENAQ is a photoswitch that restores spatially precise light responses to the blind retina. (**a**) Structure of BENAQ. Visible light converts BENAQ from the *trans* to the *cis* form and then the compound quickly relaxes back to *trans* in the dark. (**b**,**c**) MEA recordings from an *rd1* mouse retina before (**b**) and after (**c**) BENAQ treatment. Raster plots of individual RGC activity and average firing rate plots are shown. Alternating light (white) and dark (black) intervals plotted at the top. (**d**,**e**) Average *rd1* retinal firing rate in the dark and light before (left) and after (right) BENAQ treatment (n = 16 retinas). Average WT retinal firing rate in the dark (**e**, left) (n = 8 retinas). Data are mean ± SEM. (**f**) LRI value distributions for RGCs from untreated (black) (median LRI = 0.00) and BENAQ-treated (green) *rd1* retinas (median LRI = 0.51, p < 0.001, rank sum test). (**g**) MEA recording of a BENAQ treated *rd1* mouse retina stimulated with 100 ms white light flashes every 15 seconds. (**h**) White light intensity – response curve for BENAQ treated *rd1* retinas (n = 5 retinas). Light intensity threshold for driving RGC activity = 7 × 10^13^ photons/cm^2^/sec. Data are mean ± SEM, n = 5 retinas. (**i**) *Rd1* retinal light response to targeted illumination of electrode E4 with a 120 μm-diameter light spot. Only electrode E4 (red) recorded an increase in RGC activity in response to white light (bottom). LRI values are color-coded (scale at left) and also represented by bar height. (**j**) Targeted illumination elicits an increase in activity in stimulated RGCs and has no effect on surrounding RGCs (n = 17 cells and n = 903 cells, respectively, from seven retinas). LRI values of RGCs (black circles) as a function of distance from the target electrode, displayed in 200 μm bins. Median plus and minus the 95% confidence intervals are shown in red. See also Supplementary Table S1. (**k**) Responses of BENAQ-treated *rd1* RGCs to stimulation with light spots of increasing diameter. The light response saturates at 240 μm-diameter spot size. Data are mean ± SEM; n = 20 cells.

BENAQ (Fig. 1a) is an azobenzene photoswitch that blocks voltage-gated ion channels in a light-sensitive manner^8^,^9^. We evaluated the action of BENAQ on degenerated retinas of 3- to 6-month-old *rd1* mice, which lose nearly all rods and cones within 1 month after birth^10^. We measured the effect of white light on action potential firing by retinal ganglion cells (RGCs) recorded with a multielectrode array (MEA). Light elicited no change in the spontaneous firing of untreated *rd1* RGCs (Fig. 1b,d,e), but treatment with BENAQ enabled light to elicit a robust increase in *rd1* RGC firing rate (Fig. 1c,d,e). The firing rate of BENAQ-treated *rd1* RGCs (Fig. 1e, see Supplementary Fig. 1b) in darkness was lower than that of untreated *rd1* RGCs (Fig. 1e, see Supplementary Fig. 1a), suggesting that photomodulation of RGC activity after BENAQ treatment reduces the spontaneous hyperactivity of *rd1* RGCs. The median *rd1* RGC Light Response Index (LRI)^6^ increased from 0.00 (n = 1212 RGCs) to 0.51 after BENAQ treatment (n = 1153 RGCs, p < 0.001, rank sum test) (Fig. 1f). Brief light pulses (100 ms) were sufficient to generate a robust, transient light response (Fig. 1g). The minumum light intensity required to generate an RGC response was 7 × 10^13^ photons/cm^2^/sec (Fig. 1h); equivalent to daylight^11^ and similar to the threshold for activating retinas treated with DENAQ^6^ or expressing optogenetic tools^3^,^4^. The dynamic range of the BENAQ mediated response, from threshold to saturation, was ~3 log units of light intensity.

To determine whether BENAQ can restore spatially precise light reponses, we characterized the receptive field properties of BENAQ-treated RGCs. A 120 μm spot caused firing in RGCs whose activity was detected by a single electrode (median LRI = 0.60, n = 17 cells) but not in RGCs detected by neighboring electrodes (median LRI = 0.00, n = 903 cells; p < 0.001, rank sum test) (Fig. 1i,j; see Supplementary Table S1). The light response saturated when the spot was expanded to 240 μm (Fig. 1k), similar to the average diameter of the mouse RGC dendritic tree (∼200 μm^12^) as well as the RGC receptive field size measured by optogenetic activation of RGCs^13^. These findings suggest that RGCs can be manipulated independently with precise stimuli, a necessity for high-acuity vision restoration.

### BENAQ is potent and long-lasting

**Figure 2:**
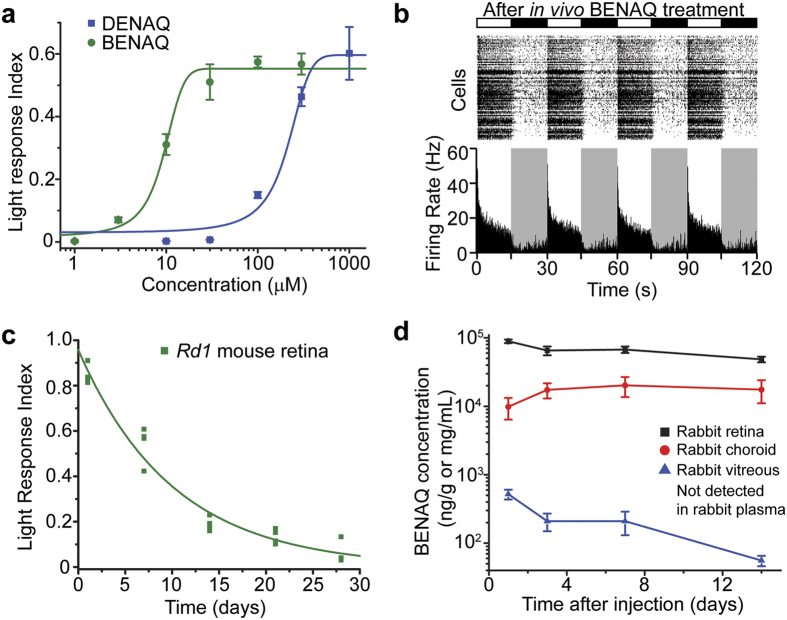
BENAQ is a potent, long-lasting photoswitch. (**a**) Retinal photosensitization dose response curves for BENAQ (green) and DENAQ (blue). BENAQ EC50 = 9.5 μM, DENAQ EC50 = 177 μM. Data are mean ± SEM, n = 5 retinas per dose. (**b**) MEA recording from an *rd1* mouse retina 7 days after intravitreal injection of BENAQ. (**c**) Time course of *rd1* mouse retinal photosensitization measured via *ex vivo* MEA recordings at various time points after a single *in vivo* intravitreal injection of BENAQ. Retinal photosensitization half-life = 7.0 days, n = 4 retinas per time point. (**d**) Pharmacokinetic characterization of BENAQ in the rabbit eye after a single intravitreal injection of 30 μM BENAQ. BENAQ retina half-life = 24 days, BENAQ vitreous half-life = 5.4 days (extrapolated based on data up to 14 days). Data are mean ± SEM, n = 6 samples from 3 animals per time point.

We next compared the potency and persistence of BENAQ and DENAQ^6^. *Ex vivo* treatment showed that BENAQ is ~20-fold more potent than DENAQ in photosensitizing the *rd1* mouse retina (EC50 of 9.5 μM and 177 μM, respectively) (Fig. 2a). *Rd1* retinas also exhibited robust light responses *ex vivo* 7 days after an *in vivo* intravitreal injection of BENAQ (half-life = 7.0 days) (Fig. 2b), in contrast to DENAQ-mediated photosensitization, which disappeared completely within 7 days after injection (half-life = 2.1 days)^6^. BENAQ-mediated photosensitization was still detectable 21 days after a single intravitreal injection (mean LRI = 0.13, n = 4 retinas, p < 0.001, t-test) but was no longer present 28 days after injection (Fig. 2c).

We carried out a further analysis of BENAQ pharmacokinetics in rabbits, which have larger eyes with morphological and functional similarities to human eyes. At various times after intravitreal injection, we collected samples of retina, choroid, vitreous humor, and blood plasma and used liquid chromatography–tandem mass spectrometry (LC-MS/MS) to measure BENAQ concentrations. Within 1 day after injection into the vitreous cavity, BENAQ became > 100-fold more concentrated in the retina than in the vitreous humor. Over the subsequent 14 days, BENAQ concentration dropped further in the vitreous (half-life = 5.4 days), but remained much more stable in the retina (half-life = 24 days) (Fig. 2d, see Supplementary Table S2). BENAQ was undetectable in rabbit plasma ( < 0.1 ng/mL) at all times after injection, indicating negligible penetration through the blood-retina barrier. These findings support the conclusion that BENAQ remains in retinal neurons for several weeks, consistent with long-term photosensitization.

### BENAQ photosensitization is specific for blind retina

**Figure 3:**
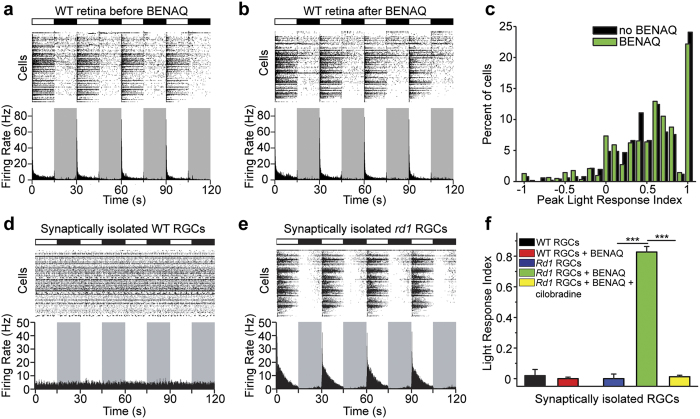
BENAQ selectively photosensitizes RGCs from degenerated but not healthy retina. (**a**,**b**) MEA recording from a WT mouse retina before (**a**) and after *ex vivo* (**b**) treatment with 300 μM BENAQ. (**c**) RGC PLRI values for WT retinas before (black) and after (green) treatment with BENAQ (n = 6 retinas, p = 0.34). (**d**,**e**) MEA recordings from BENAQ-treated pharmacologically isolated WT (**d**) and *rd1* (**e**) RGCs. (**f**) LRI values for synaptically isolated untreated (black, n = 5 retinas) and BENAQ-treated (red, n = 5 retinas) WT RGCs, as well as for untreated (blue, n = 5 retinas), BENAQ-treated (green, n = 11 retinas) and BENAQ+ cilobradine treated (yellow, n = 4 retinas) *rd1* RGCs. Data are mean ± SEM.

DENAQ confers light sensitivity on RGCs in degenerated retinas of various strains of blind mice, rats and dogs, but remarkably has no effect on healthy retinas from wild-type animals^6^,^8^. Here we report that degeneration-dependent photosensitization also applies to BENAQ. MEA recordings from wild-type retinas show a robust light response, with light onset triggering an increase in firing in some RGCs (ON-RGCs) and a decrease in others (OFF-RGCs) (Fig. 3a). BENAQ treatment caused no change in the ratio of RGCs generating On versus Off responses (Fig. 3b). To quantify On versus Off behavior, we calculated the peak light response index (PLRI), defined as the normalized change in peak firing rate upon switching from darkness to light (Fig. 3c). We found no significant difference in the distribution of PLRI values before and after BENAQ treatment (n = 6 retinas; p = 0.34, rank sum test). Thus, BENAQ appears has no effect on the response properties of the WT retina, in contrast to its effect on the *rd1* retina.

To determine whether BENAQ acts directly on RGCs, we synaptically isolated RGCs with a mixture of antagonists that block glutamate, GABA, and acetylcholine receptors (see Methods). This synaptic blocker cocktail reduced the spontaneous activity recorded from *rd1* RGCs (see Supplementary Fig. S1c). In naïve *rd1* retina, RGC light responses were almost entirely absent both before and after synaptic blockade (Fig. 3e,f). However, BENAQ treatment installed photosensitivity into RGCs, such that light elicited vigorous bursts of activity, which declined in frequency over time, presumably as a result of spike frequency adaptation mechanisms intrinsic to the RGCs (Fig. 3e,f). In contrast, BENAQ failed to support a light response in WT RGCs after synaptic blockade (Fig. 3d,f).

In order to test whether electrical coupling between RGCs and other retinal neurons or between RGCs also contributed to RGC photosensitization, we blocked gap junctions using meclofenamic acid (MFA)^14^. MFA treatment following synaptic blockade as described above strongly reduced the spontaneous activity of *rd1* RGCs (see Supplementary Fig. S1d) but did not affect *rd1* RGC photosensitization (See Supplementary Fig. S1e,f). Together, our findings suggest that BENAQ photosensitizes individual RGCs directly without requiring synaptic or electrical input from other retinal neurons, and acts only on degenerated retina.

The primary electrophysiological target for DENAQ-mediated photosensitization are HCN channels^6^, voltage-gated non-selective cation channels found in spontaneously active cells^15^. BENAQ-mediated photosensitization was likewise eliminated by cilobradine (Fig. 3f), a highly selective HCN channel blocker^16^, indicating that BENAQ and DENAQ share a common molecular target in RGCs – HCN channels.

### BENAQ exhibits low toxicity in rodent and rabbit eyes

**Figure 4:**
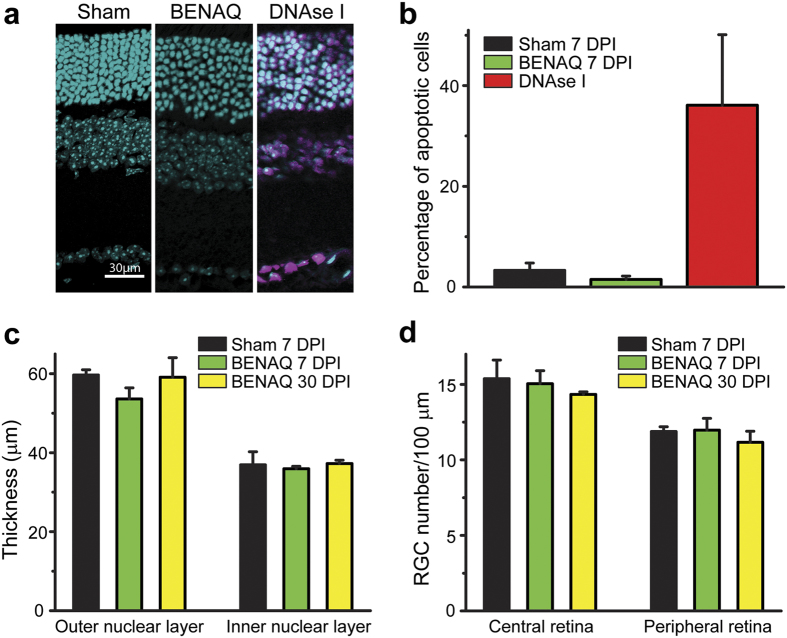
BENAQ has no apparent toxic effects on the mouse retina. (**a**) Mouse retinal cross sections 7 Days Post Injection (DPI) of sham (left), 100 μM BENAQ (center) or after DNAse I treatment (right, positive control). Cell nuclei (cyan, DAPI) and apoptotic cells (purple, TUNEL assay) are shown. (**b**) Percent apoptotic cells after sham (black) or BENAQ (green) injection at 7 DPI, with DNAse I treated retinas (red) as a positive control. Data are mean ± SEM. (**c**) Outer nuclear layer (ONL) and inner nuclear layer (INL) thickness 7 DPI of sham (black) or BENAQ (green) and 30 DPI of BENAQ (yellow). Data are mean ± SEM. (**d**) RGC count per 100 μm of retina 10 DPI of sham (black) and BENAQ (green) and 30 DPI of BENAQ (yellow). Data are mean ± SEM.

To assess the safety of BENAQ, we performed a histological analysis of WT mouse retinas after intravitreal injection of 100 μM BENAQ *in vivo*. No pathological changes were observed in the retinas of BENAQ-injected or sham-injected eyes at 7 and 30 days post-injection (DPI) (Fig. 4a). We measured apoptosis with the TUNEL assay which stains cells with nicked DNA^17^. Apoptotic cells were sparse ( < 2%) (Fig. 4a,b) and did not significantly differ in number between BENAQ- and sham-injected retinas (n = 4 retinas each, p = 0.31). In contrast, DNase I-treated retinas displayed extensive apoptosis, with 36% of retinal neurons staining positive (n = 4 retinas) (Fig. 4a,b). There was no significant difference between sham- and BENAQ-treated retinas in the thickness of the outer nuclear layer or the inner nuclear layer (Fig. 4c). Finally, there was no change in RGC density (cells per 100 μm) in the central or peripheral retina after BENAQ injection (central retina 7 DPI, n = 4, p = 0.83; 30 DPI, n = 3, p = 0.46; peripheral retina 7 DPI, n = 4, p = 0.92; 30DPI, n = 3, p = 0.44) (Fig. 4d).

We carried out additional ocular toxicity tests in Dutch Belted rabbits injected intravitreously with 30 μM or 100 μM BENAQ, doses that effectively photosensitize the retina (Fig. 2a). BENAQ was prepared with excipients (see Methods) identical to those used for clinical delivery of Ranibizumab (tradename Lucentis, Genentech/Novartis), a treatment for wet AMD. Ocular tissues were evaluated histologically at 15 days after a single injection of BENAQ. Injection of 30 μM or 100 μM BENAQ injection did not cause any pathological changes, aside from a mild inflammatory response also observed with injection of vehicle alone (see Supplementary Table S3).

## Discussion

Over the past decade, we have developed a number of photopharmacological agonists and antagonists targeting a wide variety of ion channels and neurotransmitter receptors. The first generation of engineered photoswitches, developed concurrently with optogenetics, had to be tethered to an engineered ion channel^18^. While such engineered channels robustly photosensitized retinal neurons^19^, their therapeutic potential was hampered by the need for a combination of gene therapy and intraocular photoswitch drug delivery. To overcome these limitations, we then developed a photoswitch named AAQ that targets endogenous ion channels in retinal neurons^5^. AAQ restored retinal light responses and visual behavior in blind mice but photoswitching required ultraviolet light^5^ and the compound had a very short half-life ( < 6 hours) *in vivo*. A subsequent photoswitch named DENAQ restored retinal responses to white light, but also wore off too quickly for clinical use^6^. Here, we present our latest photoswitch, named BENAQ, which we believe is more suitable than AAQ or DENAQ for potential clinical use.

BENAQ overcomes the limitations exhibited by previously tested photoswitch compounds. It is potent, long-lasting and safe in both rodent and rabbit eyes. Its action is selective for degenerated retina while having no appreciable effect on the healthy, sighted retina, raising the possibility of locally self-targeted photosensitization in patients suffering from partial vision loss due to AMD (geographic atrophy)^20^ or early stage RP^21^. BENAQ reduces the chronic hyperactivity of RGCs in degenerated retinas in darkness, an effect that may augment the quality of vision restoration. RGC hyperactivity in RP patients is thought to limit the quality of visual perception restored by retinal implants^13^,^22^. Pharmacological treatments that attenuate RGC hyperactivity enhance retinal light responses in *rd1* mice exogenously expressing a microbial opsin-derived optogenetic tool^23^. BENAQ offers a non-genetic means of suppressing intrinsic RGC hyperexcitability and imparting light-sensitivity, synergistic actions that may improve the signal to noise of neural signaling.

These features make BENAQ a favorable photoswitch candidate for preclinical development as a potential therapeutic for human use. We envision that BENAQ would be delivered through injection into the vitreous cavity of the eye. Intravitreal drug administration in humans is commonplace. For example, antiangiogenesis drugs for AMD such as Lucentis (rabinizumab) and Avastin (bevacizumab) are often administered in monthly or bimonthly injections^24^, which have proven to be safe^25^. BENAQ is soluble in the same formulation used to deliver Lucentis in the clinic and has a 10× greater lifetime in the rabbit eye as compared to Lucentis and 5× greater lifetime compared to Avastin^26^, suggesting that BENAQ may be effective with even less frequent injections. A slow release biodegradable polymer formulation^27^ may extend the release lifetime even further.

## Methods

### Chemicals

DENAQ and BENAQ were synthesized as described previously^9^. All other chemicals were purchased from Sigma-Aldrich or Tocris Bioscience.

### Animals

Retinas from WT mice (C57BL/6 J strain, Jackson Laboratory), homozygous *rd1/rd1* mice (C3H/HeJ strain, Charles River Laboratories) 3–6 months old were used in the MEA experiments. 5–6 month old Dutch Belted rabbits were used for the large animal histology and pharmacokinetic experiments. Mouse procedures were approved by the UC Berkeley Institutional Animal Care and Use Committee (IACUC) and rabbit procedures were approved by the Western Michigan University IACUC. All experiments were performed in accordance with relevant guidelines and regulations.

### Multielectrode array electrophysiology

Retinas were dissected and kept in physiological saline as previously described^6^. A solution containing (in μM) 10 AP4, 40 DNQX, 30 AP5, 10 SR-95531 (GABAzine), 50 TPMPA, 10 strychnine, 50 tubocurarine was used to pharmacologically isolate RGCs from outer retinal synaptic inputs. For extracellular recordings, a flat-mounted retina was placed ganglion cell layer down onto a multielectrode array system (MEA 1060-2-BC, Multi-Channel Systems). Retinas were treated *ex vivo* with BENAQ (300 μM unless stated otherwise) or DENAQ in the MEA chamber for 30 min, followed by a 15 min wash. In order to pharmacologically isolate RGCs, the synaptic blocker cocktail (in ACSF) was subsequently perfused for 15 min. In some experiments, gap junctions were blocked by a 15 min perfusion of 100 μM MFA after synaptic blockade. HCN channels were blocked by a 30 min perfusion of 50 μM cilobradine (in ACSF with synaptic blockers). Extracellular spikes were high-pass filtered at 200 Hz and digitized at 20 kHz. A spike threshold of 4 SD was set for each channel. Typically, each electrode recorded spikes from one to three RGCs. Principal component analysis of the spike waveforms was used for sorting spikes generated by individual cells (Offline Sorter, Plexon).

### Light Stimulation

A 100 W arc lamp (Ushio USH-103D) was used for MEA light stimulation. The photon flux equivalent for BENAQ-treated retinas was calculated using 459 nm (photoswitch absorbance peak) photon energy. The typical incident white light intensity for *rd1* and WT retinas was 2.5 × 10^15^ photons/cm^2^/sec. Neutral density filters were used to measure the light intensity-response relationship. A typical MEA stimulation protocol consisted of 10 cycles of alternating 15 sec light/dark intervals, but shorter light pulses also elicited robust responses.

### Data Analysis and Statistics

We calculated the average RGC firing rate for individual retinas in light and in darkness in some experiments. In order to normalize light-elicited changes in firing rate of individual RGCs in *rd1* retinas, we calculated the LRI = (mean firing rate in the light − mean firing rate in darkness)/(mean firing rate in the light + mean firing rate in darkness). For *rd1* mouse BENAQ ocular lifetime experiments, LRI = abs(LRI). Light-elicited changes in firing rate of individual RGCs in WT retinas were calculated as PLRI = (peak firing rate in the light − peak firing rate in darkness)/(peak firing rate in the light + peak firing rate in darkness). The first second of the light and dark intervals was used to measure the peak firing rate. Pairwise comparisons of LRI and PLRI distributions were performed using the Wilcoxon rank sum test (Matlab). All other statistical significance (p value) calculations were performed using the two tailed unpaired Student’s t test. P values are *p < 0.05, **p < 0.01, ***p < 0.001. Results with p < 0.05 were considered significant.

### Intravitreal Injections

In mouse MEA experiments, animals were anesthetized with isoflurane (2%) and their pupils were dilated with tropicamide (1%). An incision was made through the sclera, below the ora serrata with a 30 gauge needle and 2 μL of either BENAQ (2 μL of 20 mM BENAQ in 90%PBS/10%DMSO) or vehicle (sham) (2 μL of 90%PBS/10%DMSO) were injected into the vitreous with a blunt ended 32 gauge Hamilton syringe. The mice were allowed to recover for 6 hours after injection with open access to food and water in their cage. For the rabbit toxicity experiments, six Dutch Belted rabbits were injected with either vehicle alone - an aqueous solution containing 10 mM histidine HCl, 10% α, α-trehalose dihydrate, 0.01% polysorbate 20, pH 5.5 (a formulation also used for intravitreal injections of rabinizumab (Lucentis, Genentech/Roche), http://www.accessdata.fda.gov/drugsatfda_docs/label/2006/125156lbl.pdf) – or BENAQ (50 μL of 600 μM or 2 mM) dissolved in vehicle, for a final vitreal BENAQ concentration of 30 μM or 100 μM, respectively. On Day 15, all animals were euthanized and ocular tissues were collected and preserved. The ocular tissues from all animals were sent to Histo-Scientific Research Laboratories (HSRL) where they were processed, embedded in paraffin, sectioned and stained with hematoxylin and eosin (H&E). The resulting slides were evaluated via light microscopy by David S. Garlick, DVM, DACVP of HSRL.

### Pharmacokinetics

Dutch Belted rabbits were injected intravitreally with 50 μL of 600 μM BENAQ, for a final vitreal BENAQ concentration of 30 μM. Retina, choroid, and vitreous humor were collected from each eye of three rabbits/time point. Plasma was isolated from whole blood samples collected from all animals, and stored frozen. Whole blood and ocular samples were collected at 24 hours, 72 hours, 168 hours, and 336 hours after injection. An LC-MS/MS method was developed by PharmOptima, LLC and used to determine the concentration of BENAQ in the plasma, retina, choroid, and vitreous humor. No adverse abnormal observations were noted in any rabbit eyes or general health following intravitreal administration of BENAQ over the course of the study. Concentrations of BENAQ in the plasma were below the lower limit of quantitation (0.1 ng/mL). BENAQ was quantifiable in all ocular tissues out to 336 hours (14 days) postdose. The half-life was extrapolated based on the available data up to 14 days after injection.

### Cryosections

WT mice were euthanized by CO_2_ asphyxiation and cervical dislocation 7 or 30 days post intravitreal injection (DPI). For each mouse, one retina was injected with BENAQ (2 μl of 500 μM BENAQ in 90%PBS/10%DMSO), for a final vitreal BENAQ concentration of 100 μM while the other retina was injected with vehicle only (sham) (2 μl of 90% PBS/10%DMSO). For retinal cross sections, the animals were enucleated, the cornea and lens were removed, and the resulting eye-cups were fixed in 4% paraformaldehyde for 1 hour at room temperature. The tissues were then cryoprotected in 30% sucrose overnight at 4 °C and frozen in OCT compound (Tissue-TEK, Sakura) with dry-ice ethanol slurry. Retinal sections were cut (15 μm) with a Microm HM550 cryostat (Thermo Scientific) and collected on Superfrost Plus slides (Menzel–Glaser). Four sections from both retinas were collected on the same slide.

### Toxicity

Terminal deoxynucleotidyl transferase (TdT) mediated dUTP nick end labeling, or TUNEL, was used to assess apoptosis in retinal slices. Slides were treated using an *In Situ* Cell Death Detection Kit, Fluorescein (Roche) following the manufacturer’s instructions for cryopreserved tissue. Positive and negative TUNEL control sections (3000 U/ml DNAse I treated sections and sections without TdT) were included on each slide. Slides were then counterstained with 1 μM DAPI in PBS (GIBCO, pH 7.4). Fluoromount-G (Southern Biotech) was used to mount coverslips (Fisherbrand 22 × 50-1.5) onto slides. Slides were imaged on a Zeiss LSM 510 META NLO AxioImager through a 40× oil objective using DAPI and FITC filters. Images were taken at central and peripheral positions, and analyzed in ImageJ.

## Additional Information

**How to cite this article:** Tochitsky, I. *et al*. Restoring visual function to the blind retina with a potent, safe and long-lasting photoswitch. *Sci. Rep.* **7**, 45487; doi: 10.1038/srep45487 (2017).

**Publisher's note:** Springer Nature remains neutral with regard to jurisdictional claims in published maps and institutional affiliations.

## Supplementary Material

Supplementary Information
