# Supplementary material for: Restoring visual function to the blind retina with a potent, safe and long-lasting photoswitch
Source: Sci Rep. 2017 Apr 13;7:45487. doi: 10.1038/srep45487 (PMC5390669; doi:10.1038/srep45487)
Supplement: Supplementary Information [file srep45487-s1.pdf]

Restoring visual function to the blind retina with a potent, safe and long-lasting photoswitch

Authors: I. Tochitsky, J. Trautman, N. Gallerani, J. Malis, R. H. Kramer

Supplementary Information

Supplementary Materials:

Supplementary Figure S1. Effect of synaptic blockers and gap junction blockers on *rd1* RGC spontaneous activity and photosensitization.

Supplementary Table S1. Responses of BENAQ-treated *rd1* RGCs to targeted small spot illumination.

Supplementary Table S2. Pharmacokinetic parameters of BENAQ after intraocular injection in rabbits.

Supplementary Table S3. Tabulated rabbit ocular histopathologic data after intravitreal injection of vehicle or BENAQ.

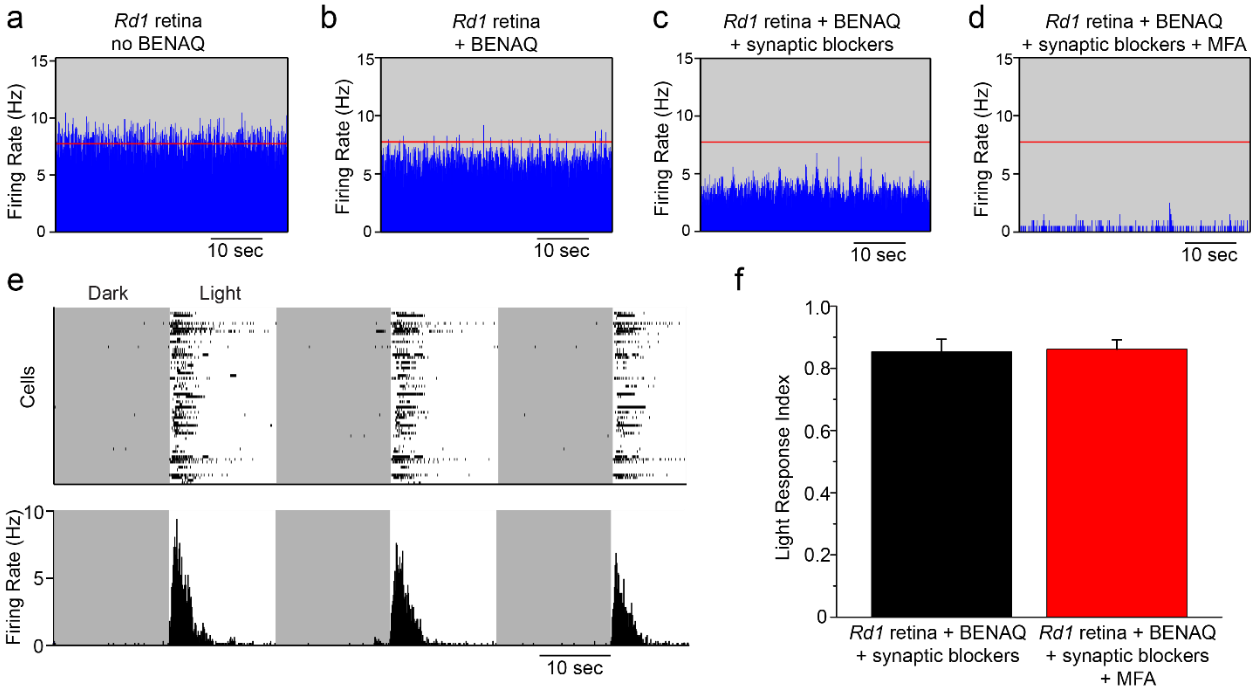

Figure S1. Effect of synaptic blockers and gap junction blockers on *rd1* RGC spontaneous activity and photosensitization.

a-d) MEA recording of average *rd1* RGC activity in the dark before BENAQ treatment (a), after BENAQ treatment (b), after application of synaptic blockers (c) and after addition of synaptic blockers + MFA (d). The average firing rate for the untreated retina is denoted by the red line.

BENAQ slightly reduces the spontaneous RGC activity, while block of synaptic transmission and gap junctions greatly reduces spontaneous RGC activity.

e) MEA recording of RGC activity from an rd1 retina after treatment with BENAQ, synaptic blockers and MFA. Even in the absence of synaptic and electrical input, RGCs treated with BENAQ remain light sensitive.

f) Quantification of BENAQ-mediated light responses in synaptically isolated *rd1* RGCs prior to and after addition of MFA (n=4 retinas, p=0.88, t-test).

**Distance (μm)**	**Median LRI**	**95% confidence interval**
Target	.60	.41 to .78
200-400	.01	0.00 to 0.03
400-600	-.01	-0.02 to 0.00
600-800	0.00	-0.01 to 0.01
800-1200	0.00	-0.02 to 0.02

Table S1. Responses of BENAQ-treated *rd1* RGCs to targeted small spot illumination.

**BENAQ rabbit pharmacokinetic parameters**	**BENAQ plasma**	**BENAQ vitreous humor**	**BENAQ retina**	**BENAQ choroid**
T_{max} (hours)	ND	24	24	168
C_{max} (ng/g or ng/mL)	ND	520	89100	20200
$t_{1/2}$ elimination (hours)	ND	130	571	ND
$AUC_{0\text{-}last}$ (ng*h/g or ng*h/mL)	ND	66200	2.09×10^7	5.74×10^6

$AUC_{0\text{-}inf}$ (ng*h/g or ng*h/mL)	ND	76600	6.08×10^7	ND

Table S2. Pharmacokinetic parameters of BENAQ after intraocular injection in rabbits.

T_{max}: time point at which maximal concentration was detected. C_{max}: maximal concentration (units: retina - ng/g, choroid – ng/g, vitreous humor – ng/mL). $t_{1/2}$: half-life. AUC: Area Under the Curve. ND: Not Determined. BENAQ not detected in plasma.

**Treatment**	**Vehicle**		**30µM BENAQ**		**100µM BENAQ**	
**Animal number**	1	2	1	2	1	2
**Organ/finding**						
**Left Eye**	Y	Y	Y	Y	Y	Y
Mononuclear cell infiltrates, bulbar conjunctiva, at limbus	1	0	0	0	0	1
Mononuclear cell infiltrates, vitreous	1	2	1	1	2	2
Mononuclear cell infiltrates, optic disc	0	1	0	0	1	1
Mononuclear cell infiltrates, optic nerve	0	2	0	0	0	0
Material, fibrillar/precipitates, vitreous	P	P	P	P	P	P
Lenticular degeneration, posterior lens, subcapsular	0	0	0	0	0	0
Retinal degeneration	0	0	0	0	0	0
Retinal detachment	0	0	0	0	0	0

**Right Eye**	Y	Y	Y	Y	Y	Y
Mononuclear cell infiltrates, bulbar conjunctiva, at limbus	1	0	0	0	0	1
Mononuclear cell infiltrates, vitreous	1	2	1	1	2	2
Mononuclear cell infiltrates, optic disc	0	1	0	0	1	1
Material, fibrillar/precipitates, vitreous	P	P	P	P	P	P
Lenticular degeneration, posterior lens, subcapsular	0	0	0	0	0	0
Retinal degeneration	0	0	0	0	0	0
Retinal detachment	0	0	0	0	0	0

Table S3. Tabulated rabbit ocular histopathologic data after intravitreal injection of vehicle or BENAQ.

Labels: Y=tissue analyzed, P=present, 0=absent, 1=minimal amount/severity, 2=mild amount/severity. Ocular tissues were evaluated histologically at 15 days after a single injection of 50 µL vehicle (Lucentis formulation saline) (n=4 eyes), 600 µM BENAQ (n=4 eyes) or 2 mM BENAQ (n=4 eyes), for a predicted final vitreal concentration of 30 µM or 100 µM BENAQ, assuming a 20x dilution in rabbit vitreous (1mL volume). No signs of retinal toxicity were observed in any of the vehicle or BENAQ treated retinas. The presence of eosinophilic material and minimal to mild mononuclear cell infiltrates (lymphocytes and macrophages) was detected in the vitreous in all vehicle-injected and BENAQ-injected eyes, most likely a result of the injection procedure itself. 50% of the eyes injected with saline or 2 mM BENAQ displayed minimal

mononuclear cell infiltrates in the bulbar conjunctiva at the limbus. This finding when present at this limited intensity is usually a spontaneous observation in Dutch Belted rabbits used in laboratory studies.
